# cryoWriter: a blotting free cryo-EM preparation system with a climate jet and cover-slip injector†

**DOI:** 10.1039/d2fd00066k

**Published:** 2022-08-04

**Authors:** Luca Rima, Michael Zimmermann, Andri Fränkl, Thomas Clairfeuille, Matthias Lauer, Andreas Engel, Hans-Andreas Engel, Thomas Braun

**Affiliations:** a Biozentrum, University of Basel Spitalstrasse 41 4056 Basel Switzerland thomas.braun@unibas.ch +41 79 7337269; b Swiss Nanoscience Institute, University of Basel 4056 Basel Switzerland; c Roche Pharma Research and Early Development, Therapeutic Modalities, Lead Discovery, Roche Innovation Center Basel, F. Hoffmann-La Roche Ltd. Grenzacherstrasse 124 4070 Basel Switzerland; d cryoWrite Ltd. Klingelbergstrasse 50 4056 Basel Switzerland

## Abstract

Electron microscopy (EM) introduced a fast and lasting change to structural and cellular biology. However, the sample preparation is still the bottleneck in the cryogenic electron microscopy (cryo-EM) workflow. Classical specimen preparation methods employ a harsh paper-blotting step, and the protein particles are exposed to a damaging air–water interface. Therefore, improved preparation strategies are urgently needed. Here, we present an amended microfluidic sample preparation method, which entirely avoids paper blotting and allows the passivation of the air–water interface during the preparation process. First, a climate jet excludes oxygen from the sample environment and controls the preparation temperature by varying the relative humidity of the grid environment. Second, the integrated “coverslip injector” allows the modulation of the air–water interface of the thin sample layer with effector molecules. We will briefly discuss the climate jet’s effect on the stability and dynamics of the sample thin films. Furthermore, we will address the coverslip injector and demonstrate significant improvement in the sample quality.

## Abstract 2

A microfluidic sample preparation method for TEM, which entirely avoids paper blotting and allows the passivation of the air–water interface during the preparation process, is presented.

## Graphical abstract

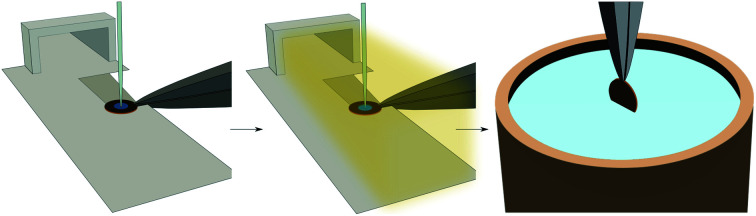

## 1 Introduction

Electron cryo-microscopy (cryo-EM)^1^ has matured into a powerful technique to study the molecular architecture of proteins close to or at atomic resolution. The success of the so-called single-particle approach is based on improved illumination systems, direct electron detectors, and data processing algorithms. However, cryo-EM sample preparation is still limiting in the cryo-EM workflow.

Several obstacles must be overcome during the sample preparation for single particle analysis by cryo-EM. First, the protein particles must be produced at sufficient quality. Production often involves over-expression systems and lengthy, harsh purification strategies. Next, the protein particles must be prepared for cryo-EM. A vitrification step is needed to keep the sample close to physiological conditions to conserve the sample’s structure in the high vacuum of the transmission EM and minimize radiation damage. Thereby, the protein particles are embedded in a thin layer (<50 nm) of a buffer. In the classical preparation methods, extensive filter paper blotting is employed to generate a thin water film, subsequently plunged into liquefied ethane to rapidly cool the specimen down for vitrification.

During the sample preparation for cryo-EM, the protein faces at least two harsh conditions. First, the paper blotting provides powerful shear forces^2^ and an extensive interface for unspecific interactions with the particles. More than 99.9% of the protein particles are lost.^3^ Second, the thin sample layer’s air–water interface (AWI) offers a harmful surface for the protein particles.^4–6^ The air provides an unphysiological, hydrophobic environment. Unfortunately, the diffusion times of the proteins from the bulk-phase to the AWI are less than 1 ms. Every collision with the interface increases the risk (i) of inducing the preferred orientation, which harms subsequent data processing to obtain a 3D model, (ii) of disintegrating protein complexes, (iii) of causing local protein disorder or complete particle denaturation, and (iv) of the oxidation of the protein’s side chains, such as aromatic amino acids, with downstream structural and functional effects.

Different approaches have been presented to avoid the paper blotting step in recent years. The initial motivations behind these developments were diverse. Some focused on automation and reduced sample consumption towards high throughput cryo-EM. Examples are ink-jet printing technology in combination with self-blotting grids (Spotiton),^7–10^ or pin-writing in combination with a cryogen jet, which also allows the vitrification of autoloader grids (VitroJet).^11^ Other groups focused on time-resolved cryo-EM developing methods for the fast sample application and subsequent vitrification. Examples are the combination of microfluidic mixing devices and sprayers,^12^ or nebulizers.^13^ Another focus was the total sample preparation for the visual proteomics of lysed single cells. Thereby, the sample handling and dispensing is accomplished by the same microcapillary.^14–17^

Also, different strategies to minimize the negative effects of AWI have been discussed and tested. Faster freezing times can minimize the number of collisions of the protein particles with the AWI, minimizing the probability of the negative effects.^13,18^ Using protecting covers, such as affinity grids,^19,20^ streptavidin 2D crystals,^21^ hydrophobin HFBI layers^22^ or graphene-based layers,^6,23–25^ can stabilize the proteins by avoiding one AWI and keep the proteins away from the second interface. Last but not least, the use of surface-active substances, such as detergent molecules, cover the AWI, therefore protecting the protein particles from the AWI.^26–28^ However, the detergents must be carefully selected before cryo-EM sample preparation, so as not to interfere with the protein’s structure.

Recently, we presented microfluidic methods and an instrument that entirely avoids paper blotting and only requires a few nL of total sample volume (including priming) to prepare negative stain and cryo-EM specimens.^3,14,15^ Notably, the system allows the direct combination of the sample preparation with different modules, such as a protein isolation module,^29^ single-cell lysis module,^16^ or buffer conditioning module.^15^ All these modules are interconnected with the same microfluidic nozzle. Therefore, the sample is transported only minimal distances, minimizing sample loss by unspecific binding to the nozzle interfaces and dilution by Taylor dispersion. Furthermore, the system allows unprecedented control and monitoring of the sample preparation process. The precise control during sample preparation and the avoidance of paper blotting are key advantages of our machine, called the cryoWriter.

However, the sample is still exposed to the harmful AWI. Although minimizing the preparation time can outrun the negative effect for some proteins,^18^ other particles are still negatively affected. Covering the interface with protective molecules can help minimize the negative consequences for proteins interacting with the AWI. Here, we present the upgraded cryoWriter system with a so-called climate jet and cover-slip injector, which allows us to charge the interface with effector molecules during the specimen preparation.

## 2 Experiments

### 2.1 The cryoWriter instrument

**Fig. 1:**
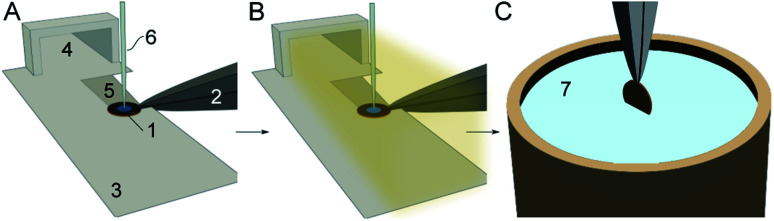
Cryo-EM sample preparation using the cryoWriter system with a climate jet and “coverslip”-injector. (A) A cryo-EM grid (1), held by a temperature-controlled tweezer (2), is placed on a “dew-point” stage (3) with good thermal contact. The climate jet (4) allows control of the temperature and the relative humidity around the grid. Note the groove (5), which allows the conditioned gas to stream on both sides of the grid (Fig. 2D). Next, the grid is primed with a thin layer of the sample by a microcapillary (6). (B) During the sample priming, a quick spray of surface-active effector molecules by a nebulizer coats the AWI. (C) After a short settling time (≈360 ms), the grid is withdrawn by temperature-controlled tweezers (2), flipped by 90° into a vertical position, and plunged in a bath of liquid ethane (7).

**Fig. 2:**
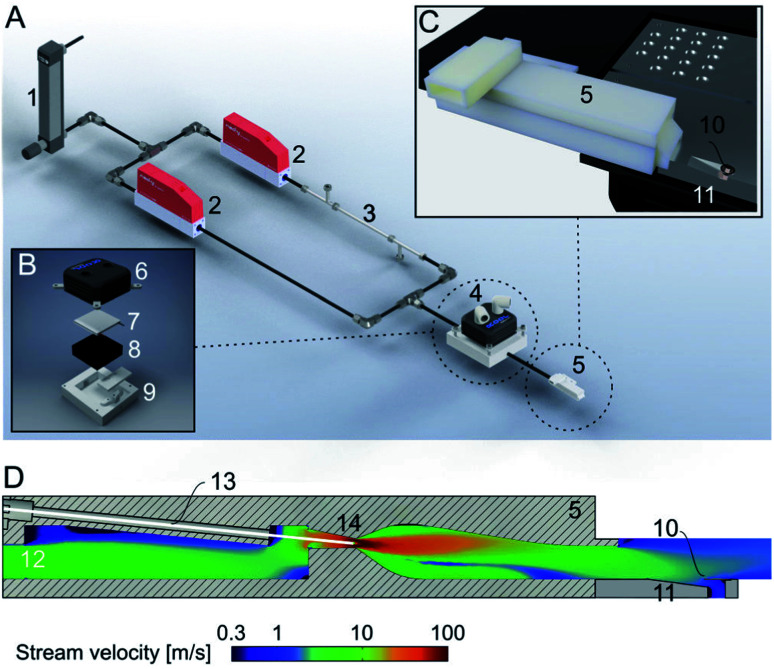
Climate jet and coverslip injector. (A) Overview. The climate jet system controls the temperature and humidity of a N_2_-stream. After passing through a pressure reducer (1), the stream is split into two lines, which are individually controlled by mass-flow controllers (2). One line is humidified by a commercial humidifying column (3). Next, the two streams are combined and enter a temperature conditioner (4), which adjusts the temperature of the two combined N_2_-streams. Finally, the gas stream enters the nozzle (5), forming a jet stream flowing over the cryo-EM grid. Note that the temperature and relative humidity are also measured in the nozzle, and two PID controls regulate the two mass-flow controllers and the temperature conditioner. (B) Details of the temperature conditioner. A water-cooled (6) Peltier element (7) efficiently heats or cools the gas stream, which flows between a metal grill (8) and plastic cap (9) for efficient heat transfer between the gas stream and the Peltier element. (C) Arrangement of the climate jet on the dew-point stage (see also Fig. 1). The nozzle (5) points toward the cryo-EM grid (10), placed with good thermal contact on top of an indentation in the temperature-controlled dew-point stage (11). (D) Details of the climate jet outlet nozzle, the N_2_-flowfield, and the nebulizer: the conditioned N_2_-stream enters a chamber (12), also hosting the sensors for the humidity and temperature measurement. A microcapillary (13), filled with a solution of effector molecules, points to the middle of a narrow restriction (14), where the stream velocity is at its maximum. The microcapillary is connected to a high-precision pump system to inject a small amount of effector molecules into the gas stream, which is then transported towards the cryo-EM grid (10) placed above an indentation in the dew-point stage (11).

The cryo-EM grid preparation principles by the cryoWriter system are described elsewhere.^14,29^ In short, the preparation is performed in 6 steps (Fig. 1): (i) a glow-discharged holey carbon film grid is horizontally positioned on a temperature-controlled stage (called the dew-point stage). Thereby, the grid is also held by temperature-controlled tweezers. Notably, a “climate jet” provides an oxygen-free environment of controlled temperature and relative humidity (see Section 2.2 and Fig. 2). The temperature of the dew-point stage is regulated at a temperature at or slightly above the dew-point of the climate jet’s gas stream. (ii) A microcapillary, connected to a high-precision pump system, is used to aspirate a liquid plug from a sample solution. Usually, a volume between 3 nL to 25 nL is used to prime the capillary with the specimen. (iii) Subsequently, the microcapillary tip is brought into proximity with the grid surface and the sample is dispensed onto the holey-carbon surface of the EM grid, as described before,^14,30^ leaving a thin film of liquid on the EM grid. (iv) During a short settling time, a controlled evaporation can be used to reduce the sample thickness to below a critical threshold and stabilize the thin film. (v) During the settling time, the climate jet is used to spray surface modulating molecules over the grid for 20 ms to 600 ms. For details, see the dedicated section below. (vi) Finally, the tweezers with the cryo-grid are picked up by a magnet, flipped by 90° into a vertical position, and plunged into liquid ethane.

### 2.2 Climate jet and coverslip injection

Here, we present a new addition to the cryoWriter system, the climate jet (Fig. 2). In our previous works,^14,29^ we used no temperature and humidity control. Therefore, the dew-point temperature and the temperature of the sample during preparation varied and were dependent on the laboratory’s climate. We have now implemented a climate jet, which provides a controlled temperature and relative humidity around the grid, allowing control of the sample preparation climate. Additionally, the jet allows the spraying of a short pulse of surface modulating molecules over the grid after sample priming, during the reaspiration of surplus liquid, and before the vitrification of the specimen.

Fig. 2A shows the principles of the climate jet. First, two N_2_-streams, one humidified by a tube humidifier and the other dry, are mixed using two mass-flow controllers. Next, in a temperature control block housing a Peltier element, the temperature of the combined gas stream is regulated. Finally, a nozzle directs the gas stream towards the cryo-EM grid, which is mounted on a groove, allowing the gas to stream along both faces of the grid. Notably, the nozzle includes (i) two sensors, measuring the gas stream’s temperature and relative humidity, and (ii) a spray generator, which nebulizes surface modulating molecules. The sensors’ information is used to control the temperature and the mixing ratio of the dry and humidified N_2_-streams by two PID loops. Together, they control the climate around the grid. Note that the grid’s temperature is maintained close to the dew-point temperature, providing a microclimate to fine-tune the thin-film’s thickness before vitrification.

The integrated nebulizer or spray allows the injection of modulator molecules into the gas stream. Ideally, these molecules build up a protective layer at the AWI and prevent the adhesion or denaturation of the sample proteins at the interface. The injection time, amount, and effector concentrations are critical for the proper formation of the protective layer. Last but not least, the physicochemical nature of the effector molecules may be varied depending on the needs of the sample. Note that the effector molecules do not need to be water soluble.

### 2.3 Climate jet outlet design and FEM simulation

The climate jet’s outlet was designed with Fusion 360 and 3D printed in PETG. The capillary for injecting effector molecules into the gas flow has an outer diameter of 360 μm and an inner diameter of 180 μm. To validate the flow properties, a finite element simulation (FEM) was performed with COMSOL Multiphysics 5.4 using the turbulent flow k-ε single-phase flow module. The material properties were taken from COMSOL, and the mesh was physically controlled with a coarse element size.

### 2.4 Testing the cryoWriter system

For initial tests of the modulator molecule injection into the gas stream of the climate jet, an octyl glucoside (OG) solution at its critical micelle concentration (CMC) was used. 150 nL were injected into the gas stream and thus nebulized. The injection of OG into the stream of gas starts simultaneously with the excess sample’s withdrawal. The protein sample (Chaperonin 60 from *E. coli*, Sigma, Switzerland) had a concentration of 0.4 mg mL^−1^ in Tris buffer (35 mM Tris, 7 mM KCl, 7 mM MgCl_2_, pH 7.5). The other test sample was pure water (water sterile-filtered, Sigma, Switzerland). Grids (R 2/2, Quantifoil, Germany) were glow discharged (air, 45 s, 100 W, 0.4 mbar) before sample application. Data was acquired on a FEI Talos (200 kV, FEG, Ceta CMOS).

#### 2.4.1 Comparison with a classical preparation

The cryoWriter grids prepared for the comparison between the cryoWriter and the classical sample preparation were prepared with the gas jet only and without using the injection system. The classically prepared grids were made with a FEI Vitrobot Mark IV. The protein sample was a type IV ABC exporter with a concentration of 0.8 mg mL^−1^ in HEPES buffer (25 mM HEPES, 100 mM NaCl, 0.001% LMNG, pH 7.5). Data was acquired on a FEI Polara (300 kV, FEG, Gatan K2).

## 3 Results and discussion

### 3.1 Thin-film formation and the effect of the climate jet

Before vitrification, a thin-film with a thickness of about 20 nm to 60 nm of the buffer containing the biological sample must be established. Unfortunately, this process is notoriously difficult, and thin-films with a thickness of 10 nm to 80 nm are inherently unstable,^31–34^ except if fresh ultra-pure water is used.^33^ The mechanistic details of thin-film formations of aqueous solutions depend on the process and the chemical–physical properties of all involved components and interfaces. Note that for thin films, the forces between the two interfaces interact and lead to a distance-dependent disjoining pressure, which is defined as the positive or negative force between the interfaces normalized by the interface area. Below the critical thickness, most thin-films are meta-stable and a dewetting process takes place, resulting in thicker droplets. The dewetting process initiates with the nucleation of the thin channel, then rapidly enlarges, leading to droplets too thick for high-resolution cryo-EM. This process usually takes place in a fraction of a second.

The classical approach for an initial thin-film formation is extensive paper blotting. This is a harsh, not well-controlled approach, leading to massive shear stress acting on the protein particles.^2^ Moreover, >99% of the precious protein particles are lost during this step.^3,17^ Recently, we presented a blotting-free method for cryo-EM grid preparation. A thin layer was written onto the grid using a microcapillary, and slight evaporation was used to stabilize the sample layer below the critical thickness long enough before vitrification (Fig. 1). A drawback of this system was that the preparation temperature depended on the dew-point temperature of the laboratory. We now implement a climate jet, which generates an oxygen-free environment of precisely defined temperature and humidity around the grid. Therefore, we can control the dew-point temperature and the cryo-EM grid’s temperature during the preparation. Fig. 2 gives an overview of the climate jet, which consists of three main parts. First, two mass-flow controllers mix a dry and a humidified N_2_-stream to control the relative humidity. Second, a temperature control block adjusts the temperature of the combined gas streams. Finally, the nozzle directs the climate jet towards the cryo-EM grid. This nozzle also hosts temperature and humidity sensors needed for feedback to the mass flow controllers and the temperature adjustment block. Notably, the nozzle also hosts a restriction, leading to high stream velocities (≈108 m s^−1^), as revealed by the finite element simulation. Here, a microcapillary allows the injection of surface modulating molecules, which are nebulized and transported towards the cryo-EM grid. A movie of the visible spray above the grid is shown in Fig. 2 of the ESI.† Note that the gas stream transports the nebulized effector molecules towards both interfaces of the EM-grid.

The climate jet influences the evaporation at the grid interface. Fig. 1 of the ESI† shows the maximal evaporation amount’s dependence on the jet stream velocity and the temperature offset from the dew-point. Note that this estimate assumes that the grid temperature stays constant throughout the process. This is not the case, since evaporation cools down the grid surface, slowing down the evaporation rate. For the usual parameters we use with the cryoWriter setup, we estimate that a layer of maximally 40 nm evaporates.

### 3.2 The harsh environment of the AWI and particle orientation

**Fig. 3:**
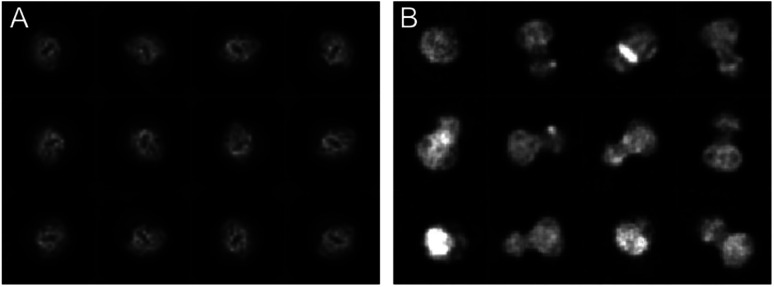
Comparison of the preparation of a membrane protein with a classical preparation system (A) and the same protein using the cryoWriter setup (B). Representative class averages are shown.

The cryoWriter setup eliminated the need for a blotting step. However, the sample is still exposed to the harsh AWI, leading to preferential orientation, or worse, harming the protein’s integrity. Our experience shows that a different priming of the specimen onto the cryo-EM grid and the absence of paper blotting can improve the particle orientation. Therefore, we have seen an improved angular distribution of the particles for different proteins when using the cryoWriter system compared to the classical plunge freezing. Fig. 3 shows a comparison of the cryo-EM preparation of a membrane protein between a classical vitrification process using the Vitrobot system and the cryoWriter without coverslip injection. Representative 2D class averages are shown for both preparations. Whereas the Vitrobot vitrification exhibits a very strong preferential orientation, the angular distribution of the protein is significantly improved using the cryoWriter system. We discuss several explanations for this observation. First, the preparation time where the protein can diffuse in the thin liquid layer is considerably shorter for the cryoWriter setup than for the Vitrobot system. Second, paper blotting is avoided, which might influence the particle state and the angular distribution. Third, the thin layer of the sample before vitrification is highly dynamic. Many interacting forces (capillary forces, van der Waals interaction, electrostatic interactions, Marangoni effects) occur, leading to the thin-layer bulk and interface liquid flow. We believe that often the latter effect dominates, since we have not seen a significant impact with different settling times before vitrification (data not shown).

#### 3.2.1 Effect of the coverslip injector

A popular approach to minimize the negative effect of the AWI on the protein particles is the introduction of surface-active molecules to the protein solution before cryo-EM grid preparation.^26–28^ Often, this is done by adding a detergent. Although this approach is beneficial, there are several drawbacks. First, a significant amount of sample is needed. Second, the detergent can interfere with the investigated proteins’ biological stability and function, and careful screening is required to select the proper additive. Third, the effector molecule needs a certain water solubility, limiting the chemical diversity used to modulate the interface.

**Fig. 4:**
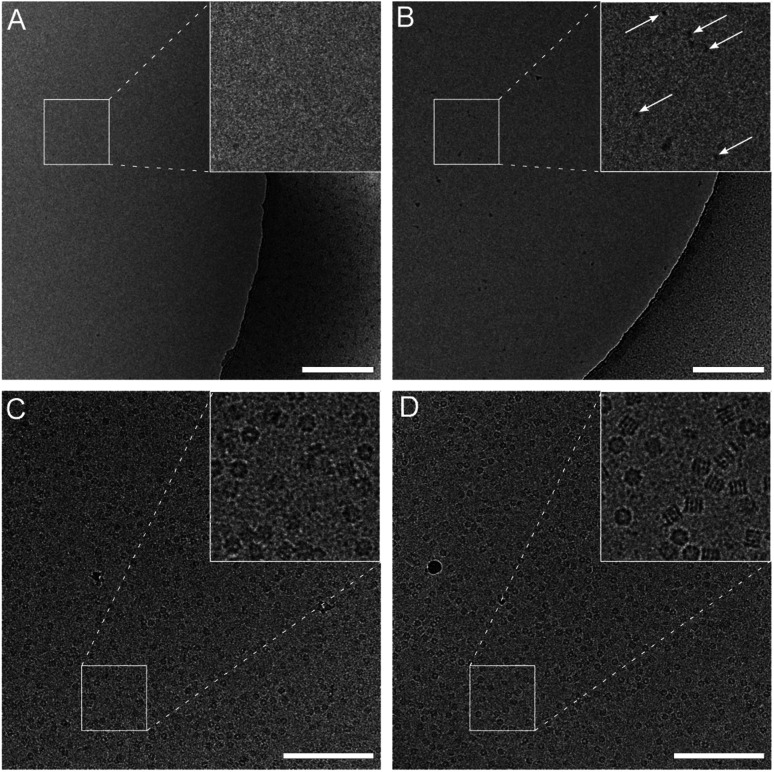
Effect of the coverslip injector with plain water (A and B) or Chaperonin 60 (C and D) as the sample. The grids were prepared without injection (A and C) or active spray (B and D). A pulse of 600 ms of an OG solution was applied simultaneously with reaspiration of the sample. See also Fig. 1 and ESI Fig. 2.† Scale bars: 200 nm.

The climate jet’s coverslip injector is not affected by these limitations. However, the effector molecule must be able to be vaporized. Here, we present experiments using the detergent OG. A pulse of 20 ms to 600 ms vaporizes the soluble detergent molecule, and the humidified N_2_-stream leads to a mass-transport of the detergent molecules towards the grid, where some of the vaporized effector droplets absorb onto the grid and release the detergent molecules. A movie with the spray is presented in Fig. 2 of the ESI.† Note that the spray is activated during the reaspiration of surplus liquid. Therefore, the protecting layer of the surface-active molecules is deposited already at the beginning of the thinning process. In the first experiment, we tested the mass transfer of OG detergent molecules of the spray onto the grid by injecting an overshoot of detergent and using water as a sample. Fig. 4A and B compare the preparation with and without the coverslip injector. The activated spray makes detergent micelles visible, whereas the negative control exhibits a clean background. We interpret this as the mass transfer of detergent molecules onto the grid, raising the detergent concentration above the CMC. Note that a higher detergent concentration was employed for this experiment than in standard preparation protocols using proteins.

We used Chaperonin 60 as a test sample to investigate the effects of the coverslip injector on the protein particle stability and orientation. Chaperonin 60 exhibits significant preferential orientation and tends to denature during cryo-EM sample preparation. For the coverslip spray, we used an OG solution at its CMC. During the preparation, the impact of the detergent molecules is readily visible by the improved sample spreading on the grid interface, as observed by the cryoWriter’s monitoring cameras. This is expected due to the decreased surface tension of the sample.

We vitrified Chaperonin 60 specimens using the cryoWriter system with and without the coverslip injector (Fig. 4C and D). Significant differences were observed between these two preparation protocols (additionally, see Fig. 3 and Table 1 of the ESI†). (i) The particle density is higher when using the coverslip injector. (ii) The ratio between the top and side views is more balanced. (iii) The fraction of intact particles is significantly higher. Moreover, (iv), the background noise in the preparation with the coverslip injector is decreased. We believe that the increased background noise originates from partially or entirely unfolded protein chains, which corroborates the decreased particle density. Note that particle unfolding is more visible in the OG treated samples. A few examples of particles that are about to unfold are presented in Fig. 4 of the ESI.†

Here, we only present one test sample (Chaperonin 60) and the effect of one effector molecule (OG). However, we generally see an improved preparation quality using the coverslip injector and other protein samples and effector molecules. We are currently investigating these effects more systematically and quantitatively.

## 4 Conclusions and outlook

We present an amended cryoWriter system, allowing the blotting-free vitrification of nanoliter-sized total sample volumes. The improved design has significant advantages compared to the device shown before by Arnold *et al.*, 2017.^14^ First, the climate jet allows the sample preparation at different temperatures and eliminates the dependency of the laboratory conditions. Second, the new spray system enables the injection of surface effector molecules immediately before the vitrification.

Although the original device did not allow the manipulation of the AWI with surface modulating molecules, the negative effect of the interface is significantly reduced for some proteins compared to classical preparation methods. The coverslip injector leads to significantly better single-particle preparations than the cryoWriter without the spray, demonstrating the beneficial effect of a coverslip injector for the sample quality. Here, we present data using the detergent OG. Currently, we are investigating other surface-active substances, including water-insoluble molecules, and their effect on the AWI to improve the protein particles’ health. These investigations include proteins of various types, including fibrilar and membrane proteins. Notably, the coverslip injector can be combined with other methods to protect the proteins from the AWI, *e.g.*, support layers.^6,19–25^ Finally, alternative protein purification strategies, such as the microfluidic protein extraction method we recently presented,^29^ also profit from the amended single-particle cryo-EM preparation method. Here, we discuss the application of the cryoWriter system to prepare single-particle samples. However, the tiny amount of consumed sample enables new kinds of experiments. For example, we combined the preparation system with a microfluidic single-cell lysis device. An adherent eukaryotic cell can be targeted in a light microscope and lysed by electroporation and the simultaneous aspiration of the cell content.^16^ Subsequently, the cell lysate can be prepared for negative stain EM^15^ and cryo-EM.^14^ Initial experiments show that the cryo-EM preparation quality of single-cell lysates is improved using the coverslip injector.

## Author contributions

TB designed the research. LR developed the climate jet and coverslip injector, and performed initial experiments. MZ is systematically testing the cryoWriter. AF, AE, HAE, ML and TC contributed new reagents/analytic tools; all authors contributed to the discussion and paper writing.

## Conflicts of interest

Thomas Braun, Andreas Engel, and Hans-Andreas Engel are coauthors of the cryoWrite Ltd. (https://www.cryowrite.com), commercializing an amended version of the cryoWrite system.

## Supplementary Material

FD-240-D2FD00066K-s001
