# Supplementary material for: cryoWriter: a blotting free cryo-EM preparation system with a climate jet and cover-slip injector
Source: Faraday Discuss. 2022 Aug 4;240:55–66. doi: 10.1039/d2fd00066k (PMC9641993; doi:10.1039/d2fd00066k)
Supplement: FD-240-D2FD00066K-s001 [file FD-240-D2FD00066K-s001.pdf]

Supplementary Information

cryoWriter: A blotting free cryo-EM preparation system with climate-jet and cover-slip injector

Luca Rima,[a] Michael Zimmermann,[a] Andri Fränkl,[b] Thomas Clairfeuille,[c] Matthias Lauer,[c] Andreas Engel,[d] Hans-Andreas Engel,[d] and Thomas Braun[a*]

[a] Biozentrum, University of Basel, Spitalstrasse 41, 4056 Basel, Switzerland. Tel: +41 79 7337269
[b] Swiss Nanoscience Institute, University of Basel, 4056 Basel, Switzerland
[c] Roche Pharma Research and Early Development, Therapeutic Modalities, Lead Discovery, Roche Innovation Center Basel, F. Hoffmann-La Roche Ltd., Grenzacherstrasse 124, 4070 Basel, Switzerland
[d] cryoWrite Ltd, Klingelbergstrasse 50, 4056 Basel, Switzerland
[*] E-mail: `thomas.braun@unibas.ch`

List of Figures

1 Theoretical maximal evaporation 2
2 Movie of the climate jet spray 3
3 Comparison of a cryo-EM grid prepared with cover slip injection and without 4
4 Examples of damaged particles. 6

List of Tables

1 Particle counts for the cryo-EM micrographs depicted in Figure 3 5

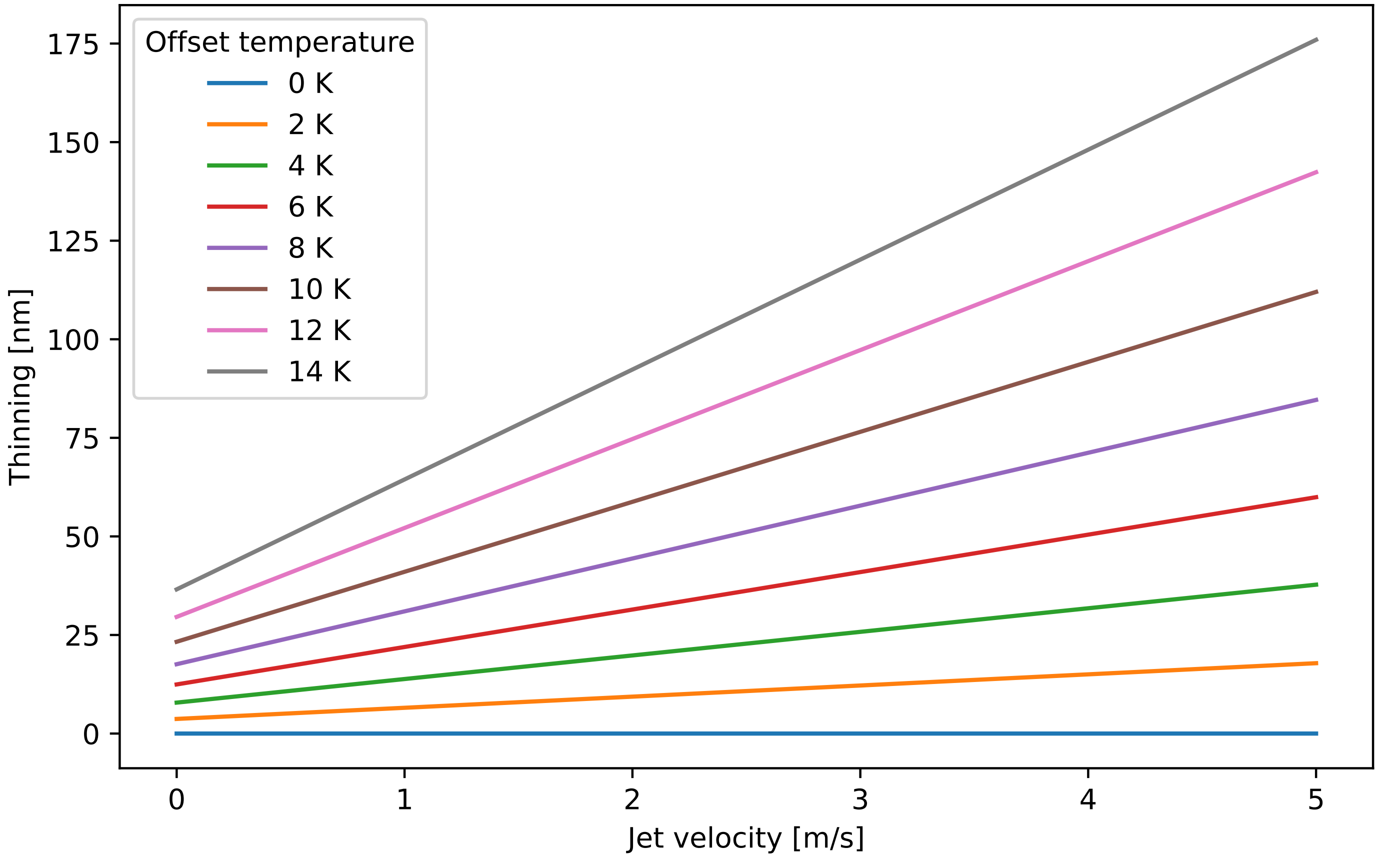

Figure 1 Maximum evaporation rate as a function of jet stream velocity and dew point offset temperature. Note that this estimate assumes that the grid temperature remains constant throughout the process. This is not the case because evaporation cools the surface of the grid, slowing the evaporation rate.

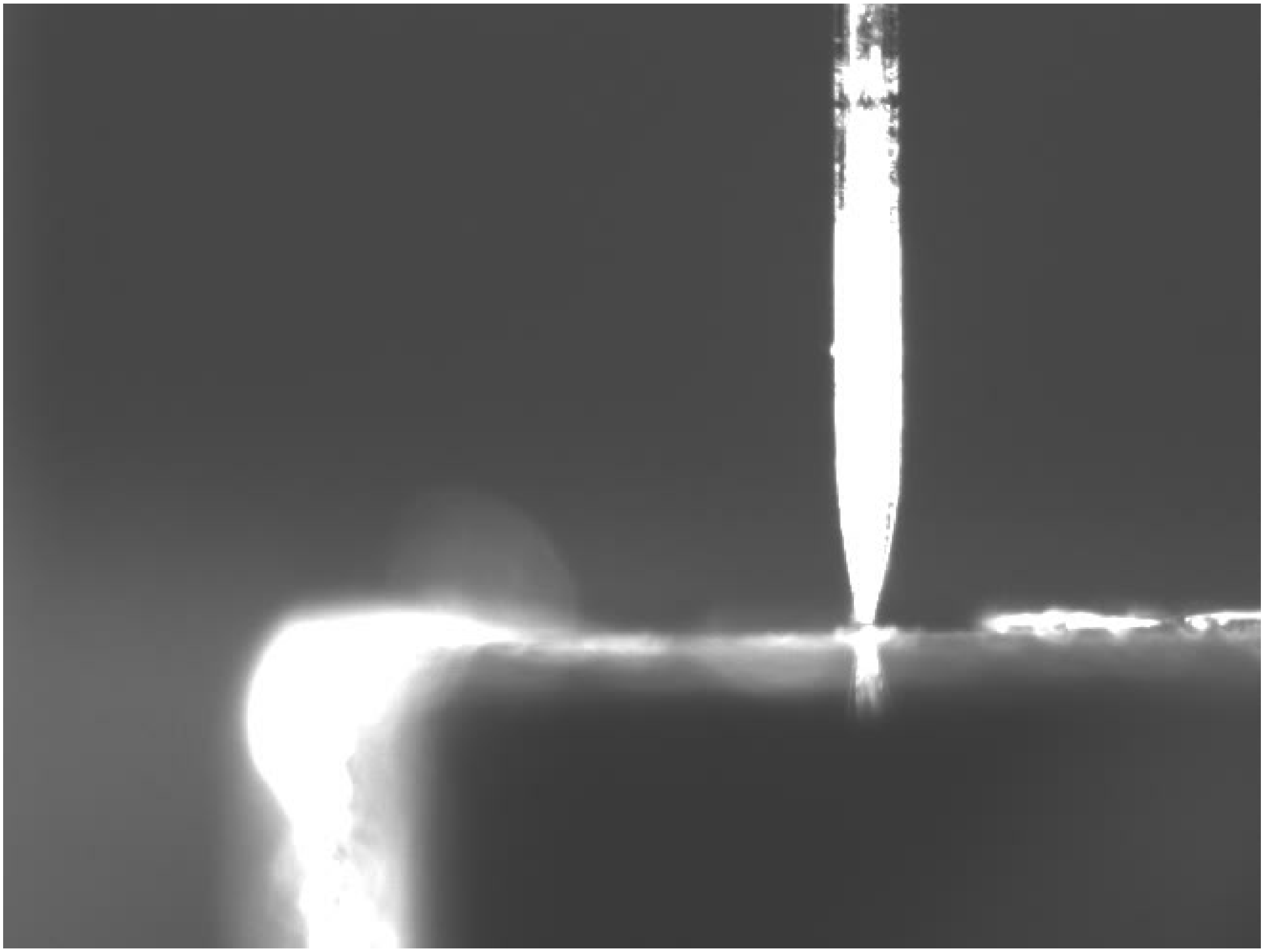

Figure 2 Movie of the climate jet spray (click to play). Shown are several injection pulses of $20\,\mathrm{ms}$ during which $50\,\mathrm{nL}$ of a detergent solution is atomized. The sprays can be recognized by the misty appearance of the entire area around the grid. The video was taken in the height plane of the grid, looking across the grid at the outlet of the climate jet nozzle.

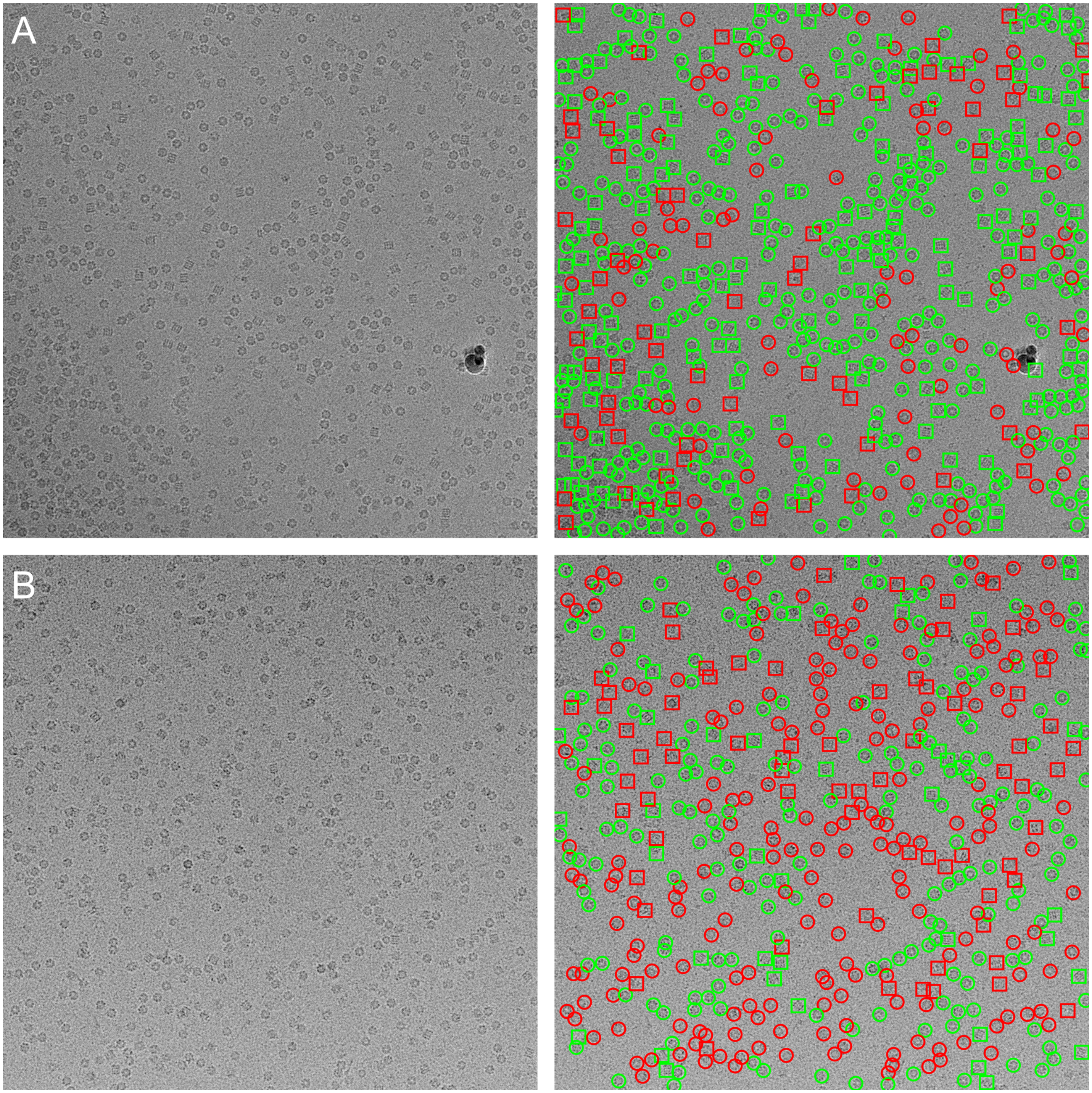

Figure 3 Comparison of a cryo-EM grid prepared with (A) cover slip injection and without (B, negative control). For each experiment, one randomly selected micrograph is used. The complete series of images can be obtained via request. The left side shows the original image. Note the preferential orientation of the particles. The right image shows an overlay indicating the particles' classification (circle: top view); square: side view). Damaged particles are indicated in red, healthy particles in green. Particles were only classified as damaged with clear visible indications. There is significantly less background noise in preparations using the cover slip injection. Consequently, particle damage is easier to detect compared to preparations without a cover slip.

Table 1 Particle counts for the cryo-EM micrographs depicted in Figure 3

	OG Injection	Negative control
Top views intact (green circles)	324	188
Side views intact (green squares)	141	39
Top views damaged (red circles)	84	191
Side views damaged (red squares)	65	68
Total particles	614	486
Total Damaged particles	149	259
Relative amount damaged particles (all)	24.3 %	53.3 %
Relative amount of damaged top views	20.6 %	50.4 %
Relative amount of damaged side views	31.6 %	63.6 %
Ratio top/side view (all)	2.0	3.5
Ratio top/side view (intact)	2.3	4.8

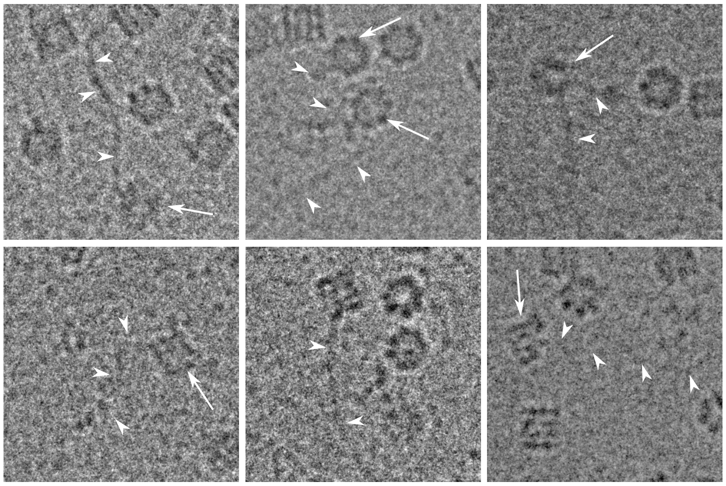

Figure 4 Examples of damaged particles. The upper row shows top views, and the lower row depicts side views. The damaged particles are indicated with white arrows; the arrowheads label putative protein parts unfolded from the damaged particle. Because of the lower background noise, all examples are selected from a cryo-EM grid prepared with the cryoWriter system using the OG coverslip injector.
